# Phosphorylated guanine nucleotide exchange factor C3G, induced by pervanadate and Src family kinases localizes to the Golgi and subcortical actin cytoskeleton

**DOI:** 10.1186/1471-2121-5-31

**Published:** 2004-08-20

**Authors:** Vegesna Radha, Ajumeera Rajanna, Ghanshyam Swarup

**Affiliations:** 1 Centre for Cellular and Molecular Biology Uppal Road, Hyderabad – 500 007 India

## Abstract

**Background:**

The guanine nucleotide exchange factor C3G (RapGEF1) along with its effector proteins participates in signaling pathways that regulate eukaryotic cell proliferation, adhesion, apoptosis and embryonic development. It activates Rap1, Rap2 and R-Ras members of the Ras family of GTPases. C3G is activated upon phosphorylation at tyrosine 504 and therefore, determining the localization of phosphorylated C3G would provide an insight into its site of action in the cellular context.

**Results:**

C3G is phosphorylated in vivo on Y504 upon coexpression with Src or Hck, two members of the Src family tyrosine kinases. Here we have determined the subcellular localization of this protein using antibodies specific to C3G and Tyr 504 phosphorylated C3G (pY504 C3G). While exogenously expressed C3G was present mostly in the cytosol, pY504 C3G formed upon Hck or Src coexpression localized predominantly at the cell membrane and the Golgi complex. Tyrosine 504-phosphorylated C3G showed colocalization with Hck and Src. Treatment of Hck and C3G transfected cells with pervanadate showed an increase in the cytosolic staining of pY504 C3G suggesting that tyrosine phosphatases may be involved in dephosphorylating cytosolic phospho-C3G. Expression of Src family kinases or treatment of cells with pervanadate resulted in an increase in endogenous pY504 C3G, which was localized predominantly at the Golgi and the cell periphery. Endogenous pY504 C3G at the cell periphery colocalized with F-actin suggesting its presence at the subcortical actin cytoskeleton. Disruption of actin cytoskeleton by cytochalasin D abolished phospho-C3G staining at the periphery of the cell without affecting its Golgi localization.

**Conclusions:**

These findings show that tyrosine kinases involved in phosphorylation of C3G are responsible for regulation of its localization in a cellular context. We have demonstrated the localization of endogenous C3G modified by tyrosine phosphorylation to defined subcellular domains where it may be responsible for restricted activation of signaling pathways.

## Background

Guanine nucleotide exchange factors (GNEFs) are components of signaling pathways that link transmembrane receptors to intracellular GTPase family members regulating a wide variety of cellular functions such as proliferation, differentiation, adhesion and apoptosis. C3G (RapGEF1) is an ubiquitously expressed GNEF for Ras family proteins that particularly targets Rap1, Rap2 and R-Ras [1-4]. It has been shown to mediate signals received from B and T cell receptor activation, growth factors, cytokines, G protein coupled receptors and also adhesion [5-15]. C3G is present in the cytoplasm in a complex with members of the Crk family of small adapter molecules. In response to stimuli, this complex is recruited to the cell membrane involving association of Crk with phosphotyrosine containing proteins like receptor tyrosine kinases, p130 Cas, IRS-1 and paxillin [16-18]. Following translocation from cytosol to cell membrane, C3G activates downstream signaling. Its activation has been shown to lead to an activation of mitogen activated protein kinase and Jun N-terminal kinase [9,12,19-21]. Studies involving overexpression of membrane targeted C3G or dominant negative forms have shown that C3G is involved in both growth suppression as well as transformation [22-24]. C3G appears to play an important role in mammalian development because C3G-/- mice die before embryonic day 7.5. These studies have shown that C3G is required for vascular myogenesis and for cell adhesion and spreading [25,26].

The C-terminus of C3G, which shows homology to CDC25, harbors the catalytic domain. The central region of C3G, which spans about 300 residues, has polyproline tracts with the ability to bind to SH3 domains of various proteins like Crk, p130 Cas, Grb2 and Hck [1,2,9,18,27]. No function has particularly been attributed to the N-terminal sequences, which do not show homology to any defined protein sequences. The non-catalytic domain of C3G has been shown to negatively regulate its catalytic activity. Deletion of the N-terminal sequences or its association through its proline sequences to Crk leads to its activation [16]. Integrin mediated cell adhesion causes tyrosine phosphorylation of C3G [28]. It has been shown that overexpression of c-Crk1 or stimulation of cells with growth hormone leads to specific phosphorylation of Y504 [21,29]. This modification results in an increase in C3G catalytic activity towards Rap1. Src and JAK have been implicated in Y504 phosphorylation of C3G. More recently we have used site – specific antibodies to show that the activation of Src family kinase Hck, leads to C3G phosphorylation on Y504 suggesting that Src family kinases can directly regulate C3G activity and function [27].

The effectiveness and precision of intracellular signal transduction depends on protein-protein interactions that regulate enzyme activity as well as subcellular localization. Cell surface receptor activation leads to assembly of adaptor protein complexes at the plasma membrane, which serve to localize guanine nucleotide exchange proteins. Earlier, both endogenous as well as exogenously expressed C3G has been shown to localize to the cytoplasm and not to associate with plasma membrane [22,30]. Since activation of C3G occurs primarily through phosphorylation at Tyr 504 and membrane recruitment, we undertook a detailed study of the subcellular localization of both exogenously expressed and endogenous Y504 phosphorylated C3G (pY504 C3G). Expression of Src family kinases or pervanadate treatment of cells, which mimics stimulation by growth factors, resulted in marked tyrosine phosphorylation of C3G at Y504. Conventional as well as optical sectioning microscopy revealed that pY504 C3G was predominantly located at the Golgi complex and the subcortical actin cytoskeleton unlike non-phosphorylated C3G, which was largely cytosolic.

## Results

### Colocalization of C3G with Hck

**Figure 1:**
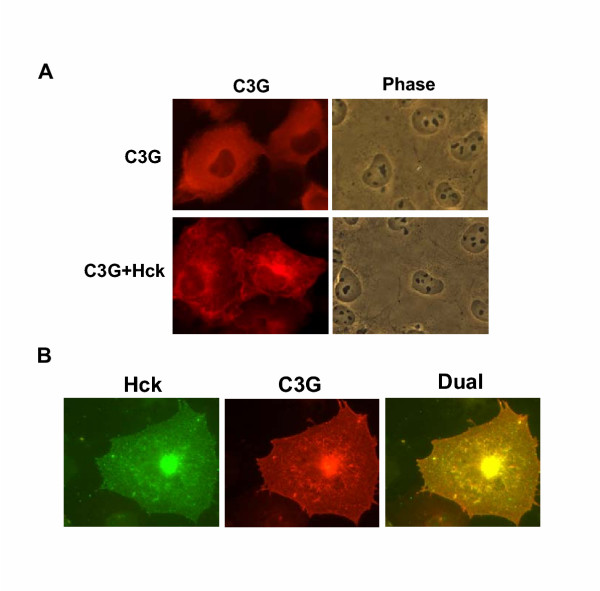
**Subcellular localization of C3G. **(A) Cos-1 cells grown on coverslip were either transfected with C3G or cotransfected with Hck and indirect immunofluorescence staining performed using anti-C3G antibodies and Cy3 conjugated anti rabbit secondaries. (B) Cells transfected with Hck and C3G were stained for both the antigens as described in Materials and Methods. Hck was visualized using FITC conjugated secondaries and C3G by Cy3 conjugated secondaries. The dual panel shows the merged image of an optical section taken using the confocal microscope where the yellow signal generated shows colocalization of the two proteins.

We have recently shown that Hck interacts with and phosphorylates C3G in vivo and we wished to determine if their interaction leads to changes in the subcellular distribution of C3G and whether pY504 C3G locates to specific subcellular domains. Cos-1 cells were transfected with C3G in the presence or absence of Hck and immunostained using anti C3G antibodies. As shown in Fig. 1A in a majority of cells, exogenously expressed C3G showed diffuse cytoplasmic staining that extended up to the plasma membrane. Variation in the level of C3G was observed among the transfected cells with weakly expressing cells showing a more prominent juxtanuclear staining. When cotransfected with Hck, most cells showed prominent staining of C3G at the plasma membrane and a juxtanuclear organelle in addition to the diffuse cytoplasmic staining. This pattern appeared similar to that seen for exogenously expressed Hck and therefore we performed colocalization studies to confirm their distribution. When coexpressed, Hck and C3G are targeted predominantly to the plasma membrane and other intracellular membranous structures (Fig. 1B). Merged images show that these two proteins colocalize in the subcellular context. Similar patterns of colocalization were observed when C3G and Hck were expressed in HeLa cells (data not shown).

### Src family kinases phosphorylate C3G and phospho-C3G localizes to the Golgi and plasma membrane

**Figure 2:**
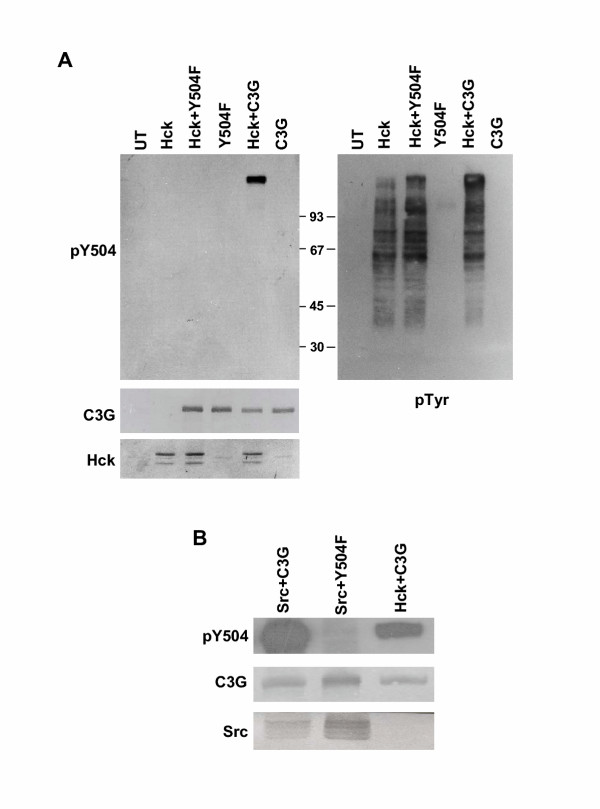
**Specificity of phosphospecific antibody, and phosphorylation of C3G on Y504 upon coexpression with Src family kinases. **Cos-1 cells were transfected with the expression constructs for Hck (A) or c-Src (B) along with C3G as indicated and western blotting of whole cell lysates was performed using the phosphospecific antibody pY504. The blots were reprobed with C3G, Hck and anti pTyr (Panel A) or Src (Panel B) to show their expression in the lysates. pY504 C3G and pTyr was detected by ECL and C3G, Hck and Src by alkaline phosphatase dependent color development. UT indicates untransfected cell lysates. Y504F is a mutant of C3G in which tyrosine 504 is replaced by phenylalanine.

To determine the subcellular distribution of phospho-C3G, which is known to be the activated form, specificity of a rabbit polyclonal phosphorylation site-specific antibody (pY504-C3G) was verified by examining its reactivity using cell lysates expressing C3G or Y504F-C3G alone or with Hck. As shown in Fig. 2A, pY504-C3G antibody recognizes only C3G when coexpressed with Hck. Neither the C3G protein expressed in itself nor the Y504F mutant coexpressed with Hck show any reactivity with this antibody suggesting that it reacts only with Y504 phosphorylated C3G. Phosphotyrosine blotting showed that a large number of cellular polypeptides are phosphorylated on tyrosine upon Hck expression, (Fig. 2A, right panel) but except for C3G none of the others show any reactivity with pY504 antibody. Y504F mutant of C3G, which shows low level of phosphorylation on other tyrosine residues is not detected by this antibody indicating its specificity towards Y504 phosphorylated C3G. Unlike Hck, whose expression is restricted to a subclass of hematopoietic cells, C3G is ubiquitously expressed and we wished to determine if other Src family kinases could phosphorylate C3G. We coexpressed C3G with an expression construct for the fusion protein c-Src-GFP and western blotting was performed using pY504-C3G antibody. As shown in Fig. 2B, in vivo, c-Src was also able to induce Y504 phosphorylation of C3G, but not that of the Y504F C3G mutant.

**Figure 3:**
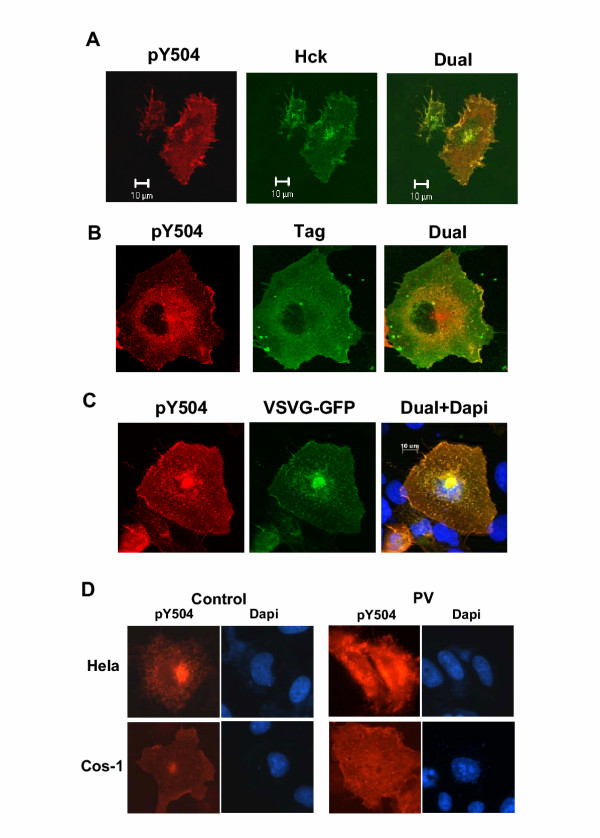
**pY504-C3G colocalizes with Hck and shows predominant Golgi and membrane localization. **(A) pY504 C3G colocalizes with Hck. Cos-1 cells transfected with Hck and C3G were stained for pY504 C3G (Cy3) and Hck (FITC) and examined using a confocal microscope. Figure shows an optical section for the individual stains as well as that of the merged (Dual) image. (B) Cos-1 cells transfected with Hck and C3G were dual labeled to detect phospho-C3G (Cy3 staining) and C3G using the Flag tag antibody (FITC staining). Panels show optical sections taken using the confocal microscope. (C) pY504 C3G is localized to the Golgi apparatus. Cos-1 cells were transfected with Hck, C3G and VSVG-GFP expression constructs and stained using pY504 primary antibody and Cy3 conjugated secondary. An optical section taken using the apotome is represented. (D) HeLa or Cos-1 cells transfected with Hck and C3G were left untreated (control) or treated with pervanadate (PV) prior to fixation and stained for pY504. Counter staining with Dapi shows cell nuclei.

We wished to determine whether C3G that colocalizes with Hck was the phosphorylated component and therefore used pY504-C3G antibodies to determine the localization of pY504 C3G in cells expressing Hck and C3G. As shown in Fig. 3A, pY504 C3G showed a staining pattern that exactly matched that of Hck with prominent staining of the plasma membrane, a juxtanuclear organelle and other intracellular membranes (3A). The prominent staining appeared to correspond with the Golgi structure and Hck has earlier been shown to localize to the Golgi [31]. In cells transfected with C3G and Hck, the pattern of pY504 C3G staining was also compared with that of total C3G as detected by the Flag tag antibody. As shown in Fig. 3B, it was observed by confocal analysis that the tag antibody detects the presence of C3G spread throughout the cytoplasm with some prominence in the juxtanuclear region and plasma membrane. The pattern suggests that the majority of the protein is cytosolic. In contrast staining for phospho-C3G was non-uniform and was particularly prominent at the Golgi and cell membrane. Colocalization of C3G with that of phospho-C3G is seen at the plasma membrane and in the juxtanuclear region. This also suggests that only a proportion of the expressed C3G is phosphorylated at Tyr504. To confirm the presence of pY504 C3G in the Golgi, we coexpressed the viral protein, VSVG-GFP known to localize to the Golgi with Hck and C3G and observed the staining pattern of pY504 C3G and that of GFP. VSVG-GFP locates predominantly at the Golgi, trans-Golgi network and also the endoplasmic reticulum and plasma membrane in a temperature-dependent manner [32]. As shown in Fig. 3C, the yellow signal generated in the dual image showed colocalization of pY504 C3G with VSVG-GFP suggesting that pY504 C3G was predominantly targeted to the Golgi complex. Unlike C3G, pY504 C3G appeared to be restricted to the plasma membrane and other intracellular membranes with particular concentration in the Golgi. When overexpressed, a large amount of C3G was present in the cytosol and we wished to determine whether cytosolic C3G does not get phosphorylated upon Hck coexpression or whether pY504 C3G in the cytosol is transient due to the action of tyrosine phosphatases. Cos-1 and HeLa cells transfected with Hck and C3G were either left untreated, or, subjected to pervanadate treatment for 10 minutes prior to fixation and stained for pY504 C3G. Pervanadate is a strong inhibitor of tyrosine phosphatases; therefore treatment of cells with pervanadate results in dramatic augmentation of tyrosine phosphorylation on cellular proteins [33]. As shown in Fig. 3D, pervanadate-treated cells showed an increase in the pY504 C3G staining in the cytoplasm suggesting that it was dephosphorylated by cytosolic tyrosine phosphatases.

### Phosphorylation of endogenous C3G and its localization to the Golgi and subcortical actin cytoskeleton

**Figure 4:**
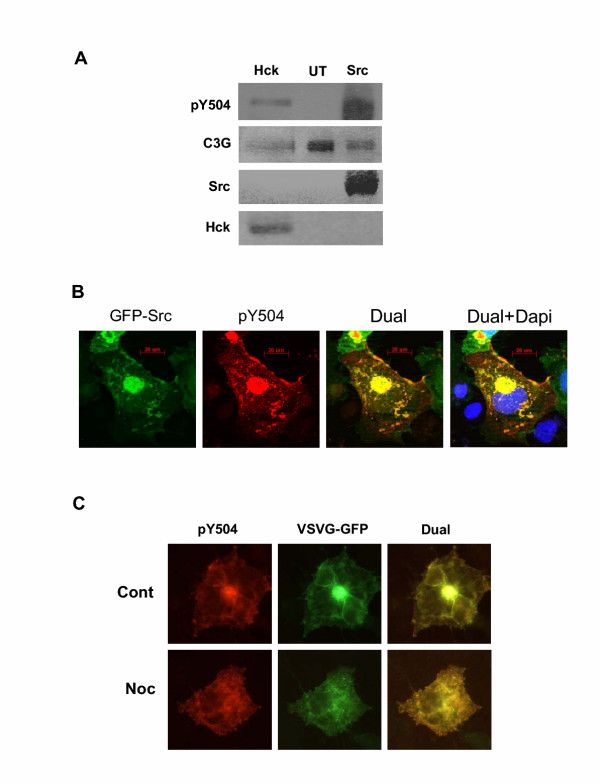
**Phosphorylation of endogenous C3G upon overexpression of Hck and its localization to the Golgi. **(A) Cos-1 cells were transfected with expression constructs as indicated and whole cell lysates used in western blotting for pY504-C3G. ECL was used for detection. Blots were reprobed to show expression of C3G and the kinases. (B) Endogenous pY504 C3G colocalizes with c-Src. Cos-1 cells were transfected with the c-Src GFP fusion protein vector and cells stained for pY504-C3G expression (Cy3). c-Src expression was visualized as GFP fluorescence. Images shown are optical sections taken using the apotome. (C) Endogenous pY504-C3G localizes to the Golgi. Cos-1 cells were transfected with Hck along with VSVG-GFP and stained for pY504 C3G. Cells were left untreated (control) or treated with nocodazole (Noc) prior to fixation as described in Methods. Panels show optical section for pY504 by Cy3 and the VSVG-GFP by GFP fluorescence.

In the above experiments phosphorylation and localization of C3G was studied using exogenously expressed protein and we wished to determine whether endogenous C3G could be phosphorylated and similarly targeted. Towards this end we checked the phosphorylation of endogenous C3G under conditions of Src and Hck overexpression or upon activation of cellular tyrosine kinases by pervanadate treatment. C3G protein is expressed as a doublet of about 140–150 kDa, which are products of two differentially spliced mRNAs [34]. Whole cell lysates were prepared from Cos-1 cells and those transfected with Hck or Src and western blotting performed using pY504 antibodies. As shown in Fig. 4A, overexpression of Src or Hck induces tyrosine 504 phosphorylation of endogenous C3G. The same blot was reprobed with C3G, Src and Hck antibodies to show their presence in the lysates. We examined the localization of endogenous phosphorylated C3G after c-Src expression and found that similar to the phosphorylated form of the exogenously expressed C3G, endogenous pY504 C3G was present predominantly at sites of c-Src localization. Intense staining of the Golgi and cell membranes was evident and merged images show colocalization of the two proteins (4B). Cells that did not express Src, did not show any phosphorylated C3G.

The localization of endogenous pY504 C3G to the Golgi was examined in Cos-1 cells transfected with Hck and VSVG-GFP. Immunostaining for pY504 C3G was seen predominantly at the cell periphery and the Golgi (Fig. 4C), which was confirmed by colocalization with VSVG-GFP. The effect of Golgi perturbing drugs on the localization of pY504 C3G was examined by treatment of cells with nocadazole for depolymerization of microtubules and concomitant Golgi fragmentation. Under these conditions pY504 C3G was detected as dispersed vesicles scattered in the cytoplasm and remained colocalized with VSVG-GFP (4C) confirming that endogenous pY504 C3G localized to the Golgi complex.

**Figure 5:**
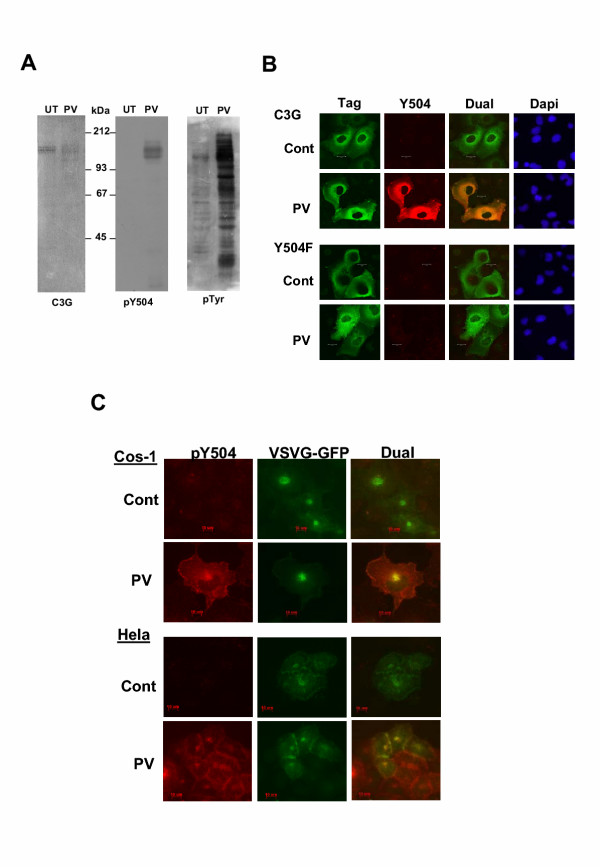
**Phosphorylation of endogenous C3G upon activation of endogenous tyrosine kinases. **(A) Cells were either left untreated (UT) or treated with pervanadate (PV) and western blotting was performed using pY504 antibody. The same blot was reprobed with C3G to show the presence of endogenous C3G in these cells. (B) Cos-1 cells on coverslips were transfected with either C3G or Y504F mutant of C3G and fixed without any treatment (cont.) or after pervanadate treatment (PV). Dual labeling was performed using the tag antibodies (stained with FITC) and pY504 antibody (stained with Cy3). Panels show optical sections obtained by confocal microscopy. (C) Cos-1 and HeLa cells grown on coverslips and transfected with VSVG-GFP were fixed without any treatment (control) or after treatment with pervanadate and stained for pY504 expression. GFP fluorescence was used to visualize the staining pattern of VSVG-GFP protein. Optical sections taken using the apotome are shown. Areas of colocalization are seen from the yellow color generated in the merged images.

The localization of pY504 C3G formed by the activation of endogenous tyrosine kinases was determined in cells treated with pervanadate, which is known to activate Src family kinases, in addition to inhibiting tyrosine phosphatases [35-37]. Pervanadate treatment results in the dramatic augmentation of phosphorylation of a large number of cellular proteins on tyrosine and therefore mimics activation of signaling pathways by growth factors [38]. While normal HeLa cells do not show any pY504 C3G, cells treated with pervanadate showed distinct presence of pY504 C3G in whole cell lysates as seen by western blotting (5A). The large number of other cellular proteins phosphorylated on tyrosine (seen upon blotting with antiphosphotyrosine antibodies), as a consequence of pervanadate treatment, do not show reactivity with pY504 antibody. In order to confirm that the signal observed upon pervanadate treatment was specific to phospho Y504-C3G, Cos-1 cells were transfected with either C3G or Y504F mutant of C3G. They were left untreated, or treated for 10 minutes with pervanadate and indirect immunofluorescence performed to observe expression of the wild type or mutant proteins as well as that of phospho-C3G. C3G, and Y504FC3G expression was monitored by staining for Flag and His tags respectively. As observed in Fig. 5B only cells expressing C3G showed intense staining for phosphoC3G while Y504F expressing cells showed no enhanced signal above that of the other non-expressing cells in the field. These results reaffirmed the specificity of the phospho-C3G antibody in detecting only Y504 phosphorylated C3G. Phosphorylated endogenous C3G staining was seen weakly in the non-expressing cells upon pervanadate treatment.

**Figure 6:**
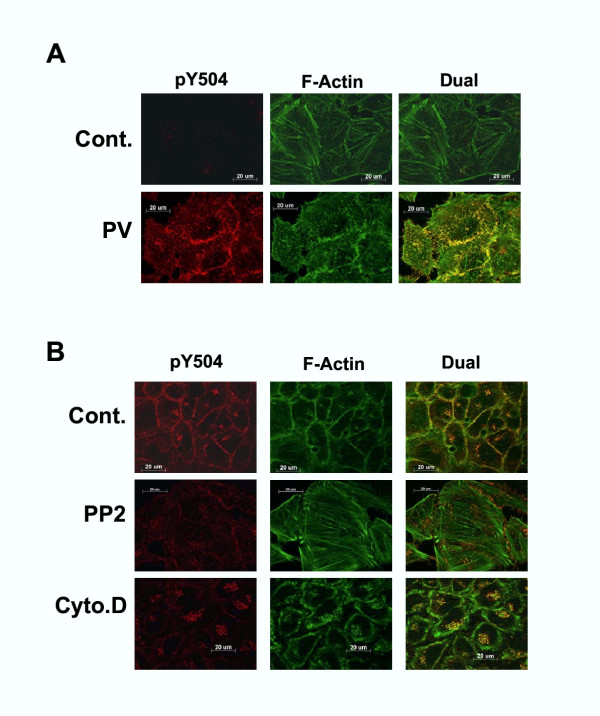
**pY504C3G localizes to the subcortical actin cytoskeleton**. (A) HeLa cells grown on coverslips were left untreated or treated with pervanadate and stained for pY504 expression using Cy3 secondaries. The coverslips were then stained with Oregon-green phalloidin to detect F-actin. (B) C3G phosphorylation requires the activity of Src family kinases and the presence of an intact cytoskeleton. HeLa cells were pretreated with PP2 or cytochalasin D as described in methods prior to pervanadate treatment. Images show the localization of pY504 C3G labeled with Cy3 and F-actin stained with oregon green. Images shown are a single optical section visualized using the apotome.

Indirect immunoflourescence was performed to determine the localization of endogenous pY504 C3G formed by the activation of intracellular tyrosine kinases. Cos-1 and HeLa cells were stimulated by pervanadate and as seen in Fig. 5C, pY504 C3G staining, which is evident only in the treated cells, localized at the cell periphery and the Golgi. Colocalization with VSVG confirmed its localization to the Golgi complex. The staining at the cell periphery appeared to match that of the subcortical actin cytoskeleton. In order to confirm this, we dual stained the cells treated with pervanadate for F-actin and found that the pY504 C3G seen at the cell periphery colocalizes with F-actin suggesting that pY504 C3G is targeted to the subcortical actin cytoskeleton upon activation of endogenous tyrosine kinases by pervanadate (Fig. 6A). It was also observed that pY504 C3G staining at the cell periphery was particularly prominent in confluent cells compared to cells that were sparsely growing in isolation suggesting that pY504 C3G is particularly enriched along cell-cell junctions. Phospho C3G also shows partial colocalization with filamentous actin known to be associated with the Golgi complex [39].

We have observed that pervanadate treatment increased tyrosine phosphorylation of endogenous C3G. Since overexpression of Src as well as Hck results in phosphorylation of C3G, it was of interest to determine whether pervanadate induced tyrosine phosphorylation of C3G was mediated by Src family kinases within the cell. Cells were treated with PP2, a specific SFK inhibitor prior to PV treatment [40]. As shown in Fig. 6B, pY504 C3G staining of cells stimulated with pervanadate was considerably reduced, but not totally abolished when they were pretreated with PP2 suggesting the possibility of other tyrosine kinase family members activated by pervanadate contributing to C3G phosphorylation. To determine whether the increase in pY504 C3G staining was dependent on the presence of an intact cytoskeleton, we observed its localization in cells treated with cytochalasin D, a reagent that effectively disrupts the actin cytoskeletal network. Under these conditions there is a collapse of cell morphology and F-actin staining shows an irregular distribution at the cell cortex. As observed in Fig. 6B, the staining for pY504 C3G in the subcortical cytoskeleton was largely absent under conditions of moderate disruption of actin organization. Under these conditions, pY504 C3G staining at the Golgi complex, which shows a more dispersed morphology appeared not to be affected.

## Discussion

C3G is involved in a variety of signaling pathways and therefore its dynamic localization under normal and activated situations may be physiologically relevant. In this study we demonstrate the limited subcellular distribution of Y504 phosphorylated C3G, which is predominantly targeted to the Golgi apparatus and the subcortical actin cytoskeleton. This localization has been substantiated by colocalization with a Golgi marker protein and F-actin respectively.

Rap1, the substrate of C3G has been localized to the Golgi, lysosomal vesicles and cortical actin cytoskeleton [41]. But, of the at least eight known exchange factors for Rap1, C3G is the only one that has been linked definitively to the tyrosine kinase signaling pathway. Src family members like Src and Hck have been shown to localize to the plasma membrane and other intracellular membranes with particular concentration in the Golgi [31,42]. When endogenous C3G was phosphorylated by overexpressed Hck or Src, the localization of pY504 C3G matched that of the kinases suggesting that they may be part of the same molecular complexes. This is also evident from the staining pattern of pY504 C3G when C3G is expressed along with Hck, which is distinctly seen in the Golgi and plasma membrane. Since exogenously expressed C3G is predominantly cytosolic, it implies that, at any given time, only a small fraction of it is phosphorylated at Y504 at the plasma membrane and the Golgi.

We observed more exogenously expressed pY504 C3G in the cytoplasmic compartment under conditions of inhibition of tyrosine phosphatases suggesting that pY504 C3G may be targeted by cytosolic tyrosine phosphatases. This regulation may help in restricting the activity of C3G to specific compartments. We observe very little endogenous pY504 C3G in the cytosol when HeLa or Cos-1 cells are treated with pervanadate, which not only inactivates tyrosine phosphatases, but also activates tyrosine kinases. It is possible that upon PV treatment endogenous C3G present in the cells is phosphorylated at the sites of location of the activated kinases. Pervanadate treatment has been shown to increase phosphotyrosine staining at the cell periphery indicating activation of kinases present in this subcellular domain [43].

Recently c-Src and Jak2 have been implicated in the phosphorylation of C3G in response to growth hormone stimulation of NIH 3T3 cells because dominant negative mutants of these kinases inhibit C3G phosphorylation [21]. It was suggested that this phosphorylation of endogenous C3G by c-Src occurs at Y504 because exogenously expressed Y504F mutant of C3G was not phosphorylated. Using a phosphospecific antibody we have directly shown the phosphorylation of endogenous C3G at Y504 upon overexpression of Hck [27] and c-Src (this report). c-Src is present in Cos-1 and HeLa cells which lack Hck. c-Src localizes to the cell membrane, focal adhesions and also to the Golgi [42,44]. Since pervanadate is a good activator of Src (36,37), it is possible that pY504 C3G seen in pervanadate treated cells is because of C3G being a Src substrate.

Adhesion dependent Src activation leads to Rap-1 activation mediated by Crk and C3G [14]. Fibroblasts lacking C3G are essentially compromised in adhesion-mediated responses [25,26]. The localization of endogenous pY504 C3G at the subcortical actin cytoskeleton therefore suggests that this may be the site of action of C3G in mediating responses to cell adhesion. Modification of C3G by phosphorylation at defined subcellular domains may be important for restricted activation of C3G mediated signaling functions in the cells. Close structural and functional relationship is known to exist between the structural elements at the cell periphery and the signal transduction machinery. Several tyrosine kinases are known to be located in adherence junctions and the kinetics of phosphorylation and dephosphorylation appears to be controlled by structural molecules at the junctions. Our observation that disruption of actin cytoskeleton results in a loss of pY504 C3G staining at the cell periphery, but not at the Golgi complex reveals an important role for cytoskeletal network in the regulation of C3G.

## Conclusions

The activity of guanine nucleotide exchange factor C3G is known to be regulated by tyrosine phosphorylation and membrane targeting. Using phospho-specific antibodies, we directly demonstrate that expression of Src family kinases or pervanadate treatment of cells induces phosphorylation of C3G on Y504. Unlike C3G, which is mostly cytosolic, pY504C3G locates to the Golgi and subcortical actin cytoskeleton. Demonstration of the localization of the active component of C3G to the Golgi and subcortical cytoskeleton provides evidence for a possible function for C3G at these cellular compartments.

## Methods

### Cell culture and treatment of cells

HeLa and Cos-1 cells were cultured in DMEM supplemented with 10% FCS. Transfections were performed on cells grown as a monolayer in either 35 mm dishes or glass coverslips using the cationic lipid DHDEAB as described [45]. Briefly, 1 μl lipid diluted in 50 μl serum free DMEM was mixed with 1 μg DNA in 50 μl serum free DMEM. The mix was kept at room temperature for 30 min to allow complex formation before adding to the cell monolayer. Cells were fed with serum 5 hrs later and harvested 24–30 hrs after transfection.

Cells were subjected to pervanadate treatment by the addition of a freshly prepared solution of pervanadate at 50 μM conc. for 10 min prior to harvesting. Pervanadate stock solution (50 mM) was prepared by mixing equal volumes of 100 mM solution of H_2_O_2_, with 100 mM solution of sodium orthovanadate. It was added to the cells within 5 mins of preparation. Golgi disruption was performed by treating the cells with 5 μg/ml of nocadazole for 30 min prior to fixation. To disrupt actin cytoskeleton, cells were treated with 1 μg/ml cytochalasin D for 20 mins. PP2 was added to cells 2 hrs before pervanadate treatment at a concentration of 10 μM to inhibit Src family kinases.

### Expression constructs

Full length human C3G cloned in pcDNA3-FLAG was kindly provided by Dr S Tanaka. Y504F mutant of C3G in which tyrosine 504 is mutated to phenylalanine cloned in a His tagged expression vector was provided by Dr M Matsuda. The wild type rat p59 Hck cDNA was cloned in the pCI plasmid (Promega) and has been described earlier [27]. Expression plasmid for vesicular stomatitis virus glycoprotein as a GFP fusion protein (VSVG-GFP) was a kind gift from Jennifer Lippincott-Schwartz [32]. c-Src-GFP expression vector expressing wild type c-Src fused to GFP at C-terminal was from Dr D L Anders [46]. Wild type human Hck cDNA cloned into pCDNA6 expression vector was a kind gift of Dr Todd Miller [47].

### Western blotting

Whole cell lysates were prepared by lysing cells directly in Laemli's sample buffer and subjected to SDS-polyacrylamide gel electrophoresis. After transfer onto nitrocellulose membranes, they were processed for western blotting using the required primary antibodies. Detection was based on either color development using alkaline phosphatase conjugated secondary antibodies or on chemiluminescence using horse radish peroxidase conjugated secondaries.

### Indirect immunoflourescence and microscopy

Cells were processed for immunoflourescence staining as described earlier [27]. The primary antibodies used were rabbit polyclonal anti-C3G (Santa Cruz Biotechnology), rabbit polyclonal anti pY504-C3G (SC-12926 R from Santa Cruz) and anti-Hck (3E9 monoclonal) made in our laboratory [48]. Dual labeling for Hck and C3G was performed by incubating the cells serially with C3G antibody, anti rabbit Cy3, monoclonal anti-Hck, and anti-mouse FITC. Cells were incubated with Oregon-green phalloidin after staining for pY504 with Cy3 to visualize F-actin. Cells transfected with vectors encoding GFP fusion proteins (GFP-Src or VSVG) were observed directly by fluorescent microscopy. Dual labeling for the C3G constructs and phospho-C3G was performed using the corresponding monoclonal tag antibodies (detected by FITC) and pY504 antibody (detected by Cy3). C3G was detected using Flag tag antibody (from Sigma) and Y504FC3G by His tag antibody (from Qiagen). Cells were examined using an Olympus microscope equipped with a cool SNAP color CCD camera. Images were captured using Image Pro Plus software. Immunoflurescence staining and colocalization was also observed using a Zeiss Axioplan 2 microscope fitted with an Apotome. The apotome (from Carl Zeiss Microimaging) is a new 3D imaging system for contrast enhancement in fluorescence microscopy. It uses structured illumination to reject signals belonging to regions of the sample that are outside the best focus position of the microscope. Images were captured using the Axiocam (Zeiss) CCD camera and processed using the Axiovision 4 software. Colocalization was also determined by observing the staining patterns using the LSM 510 Meta confocal microscope from Carl Zeiss.

## Abbreviations

GNEF – guanine nucleotide exchange factor

SFK – Src family kinase

PV – Pervanadate

pY504 C3G – Tyrosine 504 phosphorylated C3G

VSVG – Vesicular stomatitis virus glycoprotein

DMEM – Dulbecco's modified Eagle's medium

FITC – Fluorescein isothiocyanate

## Authors contributions

VR designed and carried out the experiments, analysed the data and drafted the manuscript. GS helped with designing the experiments, analyzing the data and writing the manuscript. AR provided technical help for western blotting and indirect immunoflourescence experiments.
